# Clinical outcomes of surgical embolectomy versus catheter-directed thrombolysis for acute limb ischemia: a nationwide cohort study

**DOI:** 10.1007/s11239-021-02532-1

**Published:** 2021-08-03

**Authors:** Po-Kai Yang, Chien-Chou Su, Chih-Hsin Hsu

**Affiliations:** 1 Division of Cardiology, Department of Internal Medicine, National Cheng Kung University Hospital, Dou-Liou Branch, College of Medicine, National Cheng Kung University, Yunlin, Taiwan; 2 Department of Pharmacy, National Cheng Kung University Hospital, College of Medicine, National Cheng Kung University, Tainan, Taiwan; 3 Division of Critical Care, Department of Internal Medicine, National Cheng Kung University Hospital, College of Medicine, National Cheng Kung University, 138 Sheng Li Road, Tainan, Taiwan

**Keywords:** Acute limb ischemia, Embolectomy, Endovascular therapy, Thrombolysis

## Abstract

In Taiwan, the outcomes of acute limb ischemia have yet to be investigated in a standardized manner. In this study, we compared the safety, feasibility and outcomes of acute limb ischemia after surgical embolectomy or catheter-directed therapy in Taiwan. This study used data collected from the Taiwan’s National Health Insurance Database (NHID) and Cause of Death Data between the years 2000 and 2015. The rate ratio of all-cause in-hospital mortality and risk of amputation during the same period of hospital stay were estimated using Generalized linear models (GLM). There was no significant difference in mortality risk between CDT and surgical intervention (9.5% vs. 10.68%, adjusted rate ratio (95% CI): regression 1.0 [0.79–1.27], PS matching 0.92 [0.69–1.23]). The risk of amputation was also comparable between the two groups. (13.59% vs. 14.81%, adjusted rate ratio (95% CI): regression 0.84 [0.68–1.02], PS matching 0.92 [0.72–1.17]). Age (*p* < 0.001) and liver disease (*p* = 0.01) were associated with higher mortality risks. Heart failure (*p* = 0.03) and chronic or end-stage renal disease (*p* = 0.03) were associated with higher amputation risks. Prior antithrombotic agent use (*p* = 0.03) was associated with a reduced risk of amputation. Both surgical intervention and CDT are effective and feasible procedures for patients with ALI in Taiwan.

**Supplementary Information:**

The online version contains supplementary material available at 10.1007/s11239-021-02532-1.

## Highlights

- There are limited data for comparison between catheter-directed thrombolysis and surgical intervention for acute limb ischemia in Asian population.
- There was no significant difference observed in the rates of mortality or rates of amputation between two treatment groups.
- Age and liver disease were associated with high mortality risks. Heart failure and chronic renal disease were associated higher amputation risks.

## Introduction

Acute limb ischemia (ALI) is a vascular emergency where the arterial blood supply to one or more extremities is acutely reduced in a manner that threatens the viability of a limb [1]. Without timely revascularization, the rate of limb loss can be as high as 40% with a mortality rate of 15–20% [1].

Revascularization is traditionally achieved through surgical thrombectomy or bypass surgery. With the advancement of catheter-based technologies, Catheter-directed thrombolysis (CDT) became a viable option for revascularization [2]. In Taiwan, the outcomes of acute limb ischemia has yet to be investigated in a standardized manner. In this study, we compared the safety, feasibility and outcomes of acute limb ischemia after surgical embolectomy or catheter-directed therapy in Taiwan.

## Materials and methods

### Data source

This study used data collected from the National Health Insurance Database (NHID) and Cause of Death Data between the years 2000 and 2015. The Ministry of Health and Welfare provided the two databases and the databases are maintained by the Health and Welfare Data Science Center (HWDC) in Taipei City. The NHID collects its data from the claims made to Taiwan’s National Health Insurance program, a national health insurance program that covers close to 99% of the population in Taiwan. The NHID includes a registry for beneficiaries, ambulatory care claims, inpatient claims, and prescriptions dispensed at pharmacies. All medical records in the claims data includes the diagnosis [International Classification of Diseases, Ninth Edition, Clinical Modification (ICD-9-CM) diagnosis codes] as well as the details of drugs given and procedures undertaken (e.g., date of prescription/procedure, days of supply, and National Health Insurance codes (NHICs) for all drugs and procedures covered by the program). Data in the Cause of Death Database are collected from official death certificates, data recorded on the death certificates include date of birth, sex, date of death, and cause of death. These databases can be linked with Personal identification numbers (PINs) to provide patient information such as demographics, clinical details, and information regarding cause of death.

### Study design and cohort identification

This study implemented a retrospective cohort design. The target study population were recruited from the National Health Insurance Database between 2001 and 2015. Patients who received Catheter-directed thrombolytic therapy (CDT) or surgical embolectomy (included bypass) for the first time to treat Acute limb ischemia (ALI) were included (eTable 1). The index date was defined the date of hospitalization. Patients who were under 18 years of age, had trauma related diagnoses, missing sex variables, and Length of stays (LOS) over 180 days were excluded. The present study was exempt from Institutional Review Board assessment. The Strengthening the Reporting of Observational Studies in Epidemiology checklist for cohort studies was used to write this manuscript [3].

### Outcomes and covariates

The primary outcome was defined as all-cause in-hospital mortality and the secondary outcome was defined as the risk of limb amputation (eTable 1) during the same period of hospital stay. Patient characteristics investigated in this study included age, sex, comorbidities, and co-medications. Baseline data regarding comorbidities and co-medications were gathered from a one-year look-back period prior to the index date. Detailed definitions of the comorbidities and co-medications are shown in eTables 2 and 3.

### Statistical analysis

The continuous variables were described using means and standard deviations and categorical variables were described using numbers and proportions. The Rate ratios (RR) for in-hospital mortality and amputation risk were estimated using Generalized linear models (GLM) with Poisson assumption. In order to reduce the effects of the confounding variables, we used a Propensity score (PS) matching method to adjust for confounding factors. The PS was derived from a multivariable logistic regression and the covariates included comorbidities and co-medications. Procedure timing, which defined as difference between date of procedure and date of hospitalization, are also analyzed as confounding factors. Detailed definitions regarding comorbidities and co-medications are presented in eTables 2 and 3. Patients who received embolectomies (including bypass procedures) were matched to patients who received CDT in a 1:1 ratio. All statistical analyses were done using SAS 9.4 version software (SAS Institute, Cary, NC).

## Results

The study enrolled patients admitted to hospital with ALI between 2001 and 2015 and received either CDT (n = 905) or surgical intervention (n = 4559). Patients in the CDT group were older and had a higher proportion of comorbidities compared to patients in the surgical intervention group. After propensity score matching, the two study groups were well balanced (SMD < 0.1) (Table 1).

**Table 1:** Baseline characteristics of study population

Variable	Full cohort					Matched cohort				
	CDT		Embolectomy (included bypass)		SMD^*^	CDT		Embolectomy (included bypass)		SMD
	n	% (SD)	n	% (SD)		n	% (SD)	n	% (SD)	
Total	905	100.0	4559	100.0		904	100.0	904	100.0	
Demographics										
Age, mean (SD)	71.3	13.0	69.9	13.4	− 0.10	71.2	13	70.7	12.5	− 0.04
Sex, n, %					0.21					0.02
Female	387	42.8	1494	32.8		386	43	376	42	
Male	518	57.2	3065	67.2		518	57.3	528	58.4	
Procedure timing after hospitalization, mean (SD)	1.31	1.2	1.62	1.4	− 0.24	1.28	1.2	1.32	1.3	− 0.09
Comorbidities, n, %										
Diabetes mellitus	558	61.7	2067	45.3	− 0.33	557	61.6	551	61	− 0.01
Liver diseases	70	7.7	358	7.9	0.00	70	7.7	59	6.5	− 0.05
Malignancy	151	16.7	577	12.7	− 0.11	151	16.7	146	16.2	− 0.01
Acute myocardial infarction	73	8.1	192	4.2	− 0.16	73	8.1	74	8.2	0.00
Heart failure	191	21.1	891	19.5	− 0.04	191	21.1	212	23.5	0.06
Stroke	240	26.5	1184	26	− 0.01	239	26.4	235	26	− 0.01
Ulcer disease	181	20	875	19.2	− 0.02	181	20	167	18.5	− 0.04
PAD	484	53.5	2264	49.7	− 0.08	484	53.5	500	55.3	0.04
Atrial fibrillation	110	12.2	641	14.1	0.06	110	12.2	120	13.3	0.03
Hypertension	667	73.7	3066	67.3	− 0.14	666	73.7	646	71.5	− 0.05
Hyperlipidemia	262	29	1208	26.5	− 0.05	262	29	258	28.5	− 0.01
CVD	379	41.9	1718	37.7	− 0.09	379	41.9	388	42.9	0.02
CKD/ESRD	41	4.5	138	3	− 0.08	40	4.4	28	3.1	− 0.07
Concomitant drug use, n, %										
Anticoagulant	745	82.3	3920	86.0	0.1	744	82.3	760	84.1	0.05
History of medication use, n, %										
Antiplatelets	731	80.8	3364	73.8	− 0.17	730	80.8	735	81.3	0.01
Antithrombotic agents	338	37.3	1437	31.5	− 0.12	337	37.3	330	36.5	− 0.02
RAAS inhibitors	514	56.8	2518	55.2	− 0.03	514	56.9	495	54.8	− 0.04
Beta-blocker agents	433	47.8	2102	46.1	− 0.03	432	47.8	444	49.1	0.03
CCBs	528	58.3	2680	58.8	0.01	528	58.4	499	55.2	− 0.06
Diuretics	372	41.1	2030	44.5	0.07	371	41	358	39.6	− 0.03
Statins	309	34.1	1282	28.1	− 0.13	308	34.1	306	33.8	0.00
NSAIDs	593	65.5	3122	68.5	− 0.04	593	65.6	585	64.7	0.01
Antidiabetics	545	60.2	1984	43.5	0.06	544	60.2	541	59.8	− 0.02

*SMD standardized mean difference

The rate ratios of in-hospital mortality and amputation risk in patients with ALI are summarized in Table 2. There were no significant differences in mortality risk between CDT and surgical intervention (9.5% vs. 10.68% adjusted rate ratio (95% CI): regression 1.0 [0.79–1.27], PS matching 0.92 [0.69–1.23]). The risk of amputation was also comparable between the two groups. (13.59% vs. 14.81% adjusted rate ratio (95% CI): regression 0.84 [0.68–1.02], PS matching 0.92 [0.72–1.17]).

**Table 2:** Rate ratio of mortality and amputation risk in patients with acute limb ischemia

Treatment	Case	CIR* (per 1000 persons-days)	Rate ratio (95% CI)		
			Unadjusted	Adjusted	
				Regression	PS matching
Outcome: inhospital mortality					
CDT	86	4.6	1.00	1.00	1.00
Embolectomy (included bypass)	487	4.4	0.90 (0.72–1.13)	1.00 (0.79–1.27)	0.92 (0.69–1.23)
Outcome: amputation					
CDT	123	6.6	1.00	1.00	1.00
Embolectomy (included bypass)	675	6.1	0.80 (0.70–1.02)	0.84 (0.68–1.02)	0.92 (0.72–1.17)

*Cummulative incidence rate

A multiple regression analysis for risk factor evaluation was performed (Table 3). In our study, age (*p* < 0.001) and liver disease (*p* = 0.01) were associated with higher mortality risks. Heart failure (*p* = 0.03) and chronic or end-stage renal disease (*p* = 0.03) were associated with higher amputation risks. Previous antithrombotic agent use (*p* = 0.03) was associated with a reduced risk of amputation. Gender, diabetes, hypertension, existing peripheral artery disease, stroke, atrial fibrillation, coronary heart disease, concomitant medication use, and procedure timing had no association with in-hospital mortality or amputation risk.

**Table 3:** Multiple regression analysis for in-hospital mortality and amputation risk

Variable	Outcome: In-hospital mortality			Outcome: Amputation		
	Estimates	SE	*P value*	Estimates	SE	*P value*
Treatment						
Embolectomy (included bypass)	0.12	0.15	0.43	− 0.07	0.12	0.60
CDT	0.00	−	−	0.00	−	−
Demographics						
Age	0.03	0.01	< *.0001*	− 0.01	0.01	0.22
Gender						
Male	− 0.29	0.15	0.06	0.13	0.13	0.31
Female	0.00	−	−	0.00	−	−
Procedure timing	0.25	0.19	0.24	− 0.40	0.28	0.08
Comorbidities						
Diabetes mellitus	− 0.61	0.29	0.05	0.53	0.28	0.06
Liver diseases	0.58	0.24	*0.01*	− 0.20	0.26	0.44
Malignancy	0.14	0.19	0.47	− 0.14	0.18	0.45
Acute myocardial infarction	0.02	0.29	0.95	− 0.01	0.29	0.97
Heart failure	0.26	0.21	0.21	0.40	0.18	*0.03*
Stroke	− 0.30	0.18	0.10	− 0.05	0.15	0.74
Ulcer disease	0.23	0.18	0.20	0.00	0.16	1.00
Peripheral arterial occlusive disease	− 0.36	0.17	0.05	0.05	0.14	0.69
Atrial fibrillation	0.13	0.24	0.60	− 0.43	0.29	0.13
Hypertension	− 0.18	0.20	0.38	0.19	0.17	0.27
Hyperlipidemia	0.25	0.20	0.21	− 0.05	0.16	0.77
CKD/ESRD	0.03	0.39	0.94	0.59	0.27	*0.03*
CVD	− 0.02	0.19	0.93	− 0.24	0.16	0.14
Co-medication						
Antiplatelets	0.12	0.21	0.58	− 0.10	0.17	0.56
Antithrombotic agents	0.20	0.18	0.27	− 0.36	0.16	*0.03*
RAAS inhibitors	0.14	0.18	0.45	− 0.12	0.15	0.42
Beta-blocker agents	− 0.09	0.17	0.59	− 0.09	0.14	0.51
CCBs	− 0.14	0.18	0.43	− 0.16	0.14	0.27
Diuretics	0.02	0.17	0.92	− 0.23	0.15	0.12
Statins	− 0.37	0.20	0.07	− 0.31	0.16	0.05
Non-statin lipid lowering agents	− 0.53	0.36	0.13	0.23	0.21	0.28
Antidiabetes	0.50	0.30	0.09	0.00	0.27	0.99
NSAIDs	− 0.06	0.16	0.69	0.04	0.13	0.75

## Discussion

This study is the first populational-based cohort study to investigate the outcomes of ALI in Taiwan. Our results show the following: (1) There were no significant differences between surgical intervention and CDT in regards to mortality and amputation risk, (2) older age and liver disease were associated with higher mortality risks, (3) heart failure and chronic kidney disease/end-stage renal disease were associated higher amputation risks, (4) concomitant antithrombotic agent use during hospitalization was associated with lower amputation risks.

We observed clear differences in treatment preferences based on patient characteristics. Prior to propensity weighting, patients who received surgical interventions were typically younger and had fewer comorbidities (diabetes mellitus, malignancy, previous myocardial infarction, hypertension, etc.). Patients who received CDT were more likely to have been prescribed antiplatelets, anti-thrombotics, and statins, corresponding with the higher prevalence of comorbidities in this group of patients.

There is currently very limited data on ALI outcomes. Although a number of trials comparing surgery and thrombolysis for ALI have been conducted [4, 5], there is significant degree of heterogenicity between those studies and it would be difficult for a conclusion to be made based on those studies. A meta-analysis conducted by the Cochrane Database suggested no significant differences between the two procedures in regards to limb salvage or death at 30 days [6]. However, all five trials included in the review were published before 2000. With advancement of catheter technology, the outcomes reported in those studies may not be as relevant today. In 2013, Ashraf et al. reported lower 30-day mortality and amputation rates with CDT compared to surgical intervention (mortality rates of 5.4% vs 13.2% and amputation rates of 6.5% vs 13.5% respectively) [7]. However, our study did not find a clear difference between these two parameters. Our study showed a higher prevalence of diabetes during an analysis of patient characteristics. Since the Rutherford category was not reported in our study, the severity of lower limb arterial disease and ischemia could not be evaluated in our study population. Some patients in this study may have had Rutherford III ischemia, which confers a significantly higher mortality and amputation rate.

In our study, age and liver disease were associated with a higher mortality risk. Heart failure and advanced kidney disease were associated with a higher amputation risk. These finding are mostly consistent with what has been reported in the literature in regards to risk factors for both acute and critical limb ischemia [2, 8]. However, liver disease has yet to be reported as a risk factor for mortality in limb ischemia. Chronic hepatitis B virus (HBV) and hepatitis C virus (HCV) infections are two major causes of chronic liver diseases and liver cirrhosis [9]. Taiwan is an HBV and HCV endemic country [10]. In our study, 7–8% of the study population had existing liver disease. Coagulopathy is a common finding in chronic liver disease, particular in the end stage liver disease [11]. Hemorrhagic events are an important risk factor for mortality [8]. The higher prevalence of liver disease in our study population may decrease the significance of this influence.

Duration of the diseases influenced outcomes of CDT has been reported before [12]. Therefore, procedure timing was listed as a confounding factor and adjusted by propensity score matching. Multivariable logistic regression indicated that procedure timing has no association with in-hospital mortality or amputation risk in our study population.

The findings of this study should be interpreted in light of several limitations. This study relied on discharge diagnosis codes to identify ALI events, and as such, inaccurate coding may have been a significant limiting factor in regards to this study’s inclusion criteria. In addition, previous studies have suggested a difference in thrombolytic outcomes between patients with embolic and non-embolic ischemia [13]. The NHIRD diagnosis code does not allow for the separate analysis of patients presenting with embolic and non-embolic ischemia. Besides, the information for indication of choosing one treatment modality from another are missing. CDT treatment is usually used for the management of patients who are having more underlying diseases. To reduce effects of age and comorbidities, a propensity score matching method was used to balance both groups. The database also lacks information on the affected vessels for individual patients. Newer endovascular devices, such as mechanical thrombectomies or pharmaco-mechanical thrombectomies, are not included in national health insurance system. Patients with ALI presented with different Rutherford class severities, which in turn, indicated different prognoses [14]. Detailed information on Rutherford class severity is not available in the NHIRD.

In conclusion, ALI remains a morbid condition with high risk of limb loss and death. Both surgical intervention and CDT are effective and feasible procedures for patients with ALI in Taiwan. The revascularization plan should be made carefully based on patient characteristics. Additional clinical data of good quality are needed to define longer term outcomes.

## Supplementary Information

Below is the link to the electronic supplementary material.

Supplementary file1 (DOCX 18 kb)

## Data Availability

Data are available from the National Health Insurance Research Database (NHIRD) published by Taiwan National Health Insurance (NHI) Bureau. Due to legal restrictions imposed by the government of Taiwan in relation to the “Personal Information Protection Act”, data cannot be made publicly available. Requests for data can be sent as a formal proposal to the NHIRD (http://nhird.nhri.org.tw).
