# Supplementary material for: Clinical outcomes of surgical embolectomy versus catheter-directed thrombolysis for acute limb ischemia: a nationwide cohort study
Source: J Thromb Thrombolysis. 2021 Aug 3;53(2):517–22. doi: 10.1007/s11239-021-02532-1 (PMC8904339; doi:10.1007/s11239-021-02532-1)
Supplement: Supplementary file 1 — Supplementary file1 (DOCX 18 kb) [file 11239_2021_2532_MOESM1_ESM.docx]

Appendix

eTable 1. The National Health Insurance Codes (NHIC) for catheter-directed thrombolytic therapy, bypass, surgical embolectomy, and amputation.

| Item | National Health Insurance Codes (NHICs) |
| --- | --- |
| Catheter-directed therapy | 33074B、33115B |
| Bypass | 69004B |
| Surgical embolectomy | 69001B、69002B |
| Amputation | 64022B、64023B、64024B、64025C |

eTable 2. The ICD-9-CM code for comorbidity.

| Comorbidity | ICD-9 code |
| --- | --- |
| Diabetes mellitus | 250 |
| Liver diseases | 571,572, 573 |
| Malignancy | 140-239 |
| Chronic kidney disease | 585 |
| Acute myocardial infarction | 410 |
| Heart failure | 428 |
| Stroke | 430-438 |
| Ulcer disease | 531-534 |
| Peripheral Arterial Occlusive Disease | 440-447 |
| Atrial fibrillation | 427.31 |
| Hypertension | 401-405 |
| Hyperlipidemia | 272.0-272.4, 272.9 |

eTable 3. The ATC code for co-medications.

| **Co-medication** | **ATC code** |
| --- | --- |
| Urokinase | B01AD04 |
| Alteplase (rt-PA) | B01AD02 |
| Antiplatelets | B01AC, aspirin (N02BA01, N02BA51, N02BA71) |
| Antithrombotic agents | B01AA,B01AB, B01AD (exclude B01AD02, B01AD04), B01AX,B01AE, B01AF, B01AX |
| RAAS inhibitors | C09 |
| beta-blocker agents | C07 |
| CCBs | C08 |
| Diuretics | C03 |
| Statins | C10AA, C10B |
| Non-statin lipid lowering agents | C10AB, C10AC, C10AD, C10AX |
| NSAIDs | M01A (exclude M01AX25, M01AX05, M01AX26, M01AX68) |
| Antidiabetes | A10 |
| Anti-coagulant | B01AA, B01AB, B01AE, B01AF, B01AX05 |
